# Knowledge, attitude, and practice of psoriasis patients toward their diseases: a web-based, cross-sectional study

**DOI:** 10.3389/fmed.2024.1288423

**Published:** 2024-04-10

**Authors:** Jun Tian, Lei Zhang, Xiangrong Zhao, Li Yang

**Affiliations:** ^1^Department of Dermatology, Shaanxi Provincial People’s Hospital, Xi’an, China; ^2^Shaanxi Provincial Key Laboratory of Infection and Immune Diseases, Xi’an, China

**Keywords:** knowledge, attitude, practice, psoriasis, questionnaire, cross-sectional study

## Abstract

**Objective:**

To investigate the knowledge, attitude, and practice (KAP) of psoriasis patients toward the disease.

**Methods:**

A web-based cross-sectional study was conducted among psoriasis patients who were diagnosed at the outpatient of Shaanxi Provincial People’s Hospital in March 2023. A self-designed questionnaire was administered for data collection and KAP assessment.

**Results:**

A total of 526 valid questionnaires were included, including 257 males (48.86%) psoriasis patients. Their mean KAP scores were 8.09 ± 3.60 (possible range: 0–12), 31.94 ± 4.61 (possible range: 10–50), and 51.92 ± 8.83 (possible range: 15–75), respectively. Pearson’s correlation analysis showed a positive correlation between knowledge and attitude (*r* = 0.186, *p* < 0.001), a positive correlation between knowledge and practice (*r* = 0.313, *p* < 0.001), and a negative correlation between attitude and practice (*r* = −0.181, *p* < 0.001). Moreover, structural equation model showed that medication (β = 2.74, 95% CI: 2.17, 3.32, *p* < 0.001) has significantly positive effect on knowledge. Education (β = 0.56, 95% CI: 0.31, 0.81, *p* < 0.001) and duration of psoriasis (β = 1.01, 95% CI: 0.54, 1.49, *p* < 0.001) have significantly positive effect on attitude. Knowledge (β = 1.03, 95% CI: 0.80, 1.26, *p* < 0.001) and medication (β = 4.59, 95% CI: 2.78, 6.40, *p* < 0.001) has significantly positive effect on practice, while attitude (β = −0.41, 95% CI: −0.57, −0.26, *p* < 0.001) and duration of psoriasis (β = −2.53, 95% CI: −3.49, −1.57, *p* < 0.001) exhibit significantly negative effect on practice.

**Conclusion:**

Psoriasis patients have good knowledge, positive attitude, and proactive practice toward the disease. Education, medication, duration of psoriasis might have effect on their KAP.

## Introduction

Psoriasis is a chronic autoimmune condition that causes the rapid buildup of skin cells, resulting in thick, scaly patches on the skin (1). The exact cause is unknown, but it is believed to be a combination of genetic and environmental factors (2). Stress, infections, certain medications, and weather changes can trigger or worsen flare-ups (3). Psoriasis affects around 2%–3% of the global population, occurring at any age but most commonly between 15 and 35 (4). Symptoms include red patches with silvery scales, dry skin, itching, and joint pain. While psoriasis cannot be cured, treatment aims to reduce inflammation and manage symptoms. The common treatment options include topical creams, light therapy, oral medications, and injectable drugs (5). Living with psoriasis can be difficult both physically and emotionally; therefore, counseling can be beneficial (6).

Understanding the knowledge, attitude, and practice (KAP) of individuals toward psoriasis is crucial in order to provide efficient management and support (7). Knowledge analysis reveals that many people have a limited understanding of the underlying causes and triggers of psoriasis (8). Some misconceptions include associating it with poor hygiene or contagiousness, leading to social stigmatization (9). Attitude analysis often reveals negative emotions such as embarrassment, shame, and low self-esteem, affecting the mental wellbeing of individuals living with psoriasis (10). In terms of practice, findings indicate that adherence to treatment regimens can be a challenge due to the inconvenience or side effects of medications (11). Additionally, it is common for individuals to self-manage their condition by applying various topical creams or using home remedies without consulting a healthcare professional. Therefore, a comprehensive approach is required to address these issues, such as by improving patient education programs, raising awareness to reduce stigmatization, and optimizing treatment options to enhance adherence. An improvement of KAP toward psoriasis can empower individuals to better manage their condition and improve their overall quality of life. Despite the immense importance of awareness of psoriasis among patients, a comprehensive study of KAP toward this disease is lacking. Therefore, this study aimed to assess the KAP among psoriasis patients toward the disease.

## Materials and methods

### Study design and participants

This cross-sectional study was conducted among psoriasis patients who were diagnosed at the outpatient of Shaanxi Provincial People’s Hospital in March 2023. The inclusion criteria for the study were as follows: (1) aged 18–80 years; (2) meet the diagnostic criteria (skin lesions, and their symmetrical distribution, silvery while scale, itching, and pitting, onycholysis, thickening, and discoloration of nails) for different types of psoriasis; and (3) provide informed consent and actively cooperate. The exclusion criteria for the study were as follows: (1) history of comorbid mental illness; (2) cognitive impairment; and (3) unable to respond with normal literacy. The ethical approval was waived by the ethics committee of Shaanxi Provincial People’s Hospital as this study is not involved in ethical concerns, and informed consent was obtained from all participants.

### Questionnaire

The questionnaire was designed based on the (12–14) and revised according to feedback from two senior experts. The reliability of the questionnaire was determined using Cronbach’s (α = 0.8517) based on 51 copies of the pilot questionnaire.

The final questionnaire was in Chinese and consisted of 52 items divided into four dimensions of information collection, including basic information (15 items), knowledge (12 items), attitude (10 items), and practice (15 items). For statistical analysis, scores were assigned based on the number of options for each item. For the knowledge dimension, correct responses were awarded 1 point, while incorrect or unclear answers received 0 points. The cut-off value for knowledge dimension was set at: 0–6 for poor level of knowledge and 7–12 for good level of knowledge. In the attitude and practice dimensions, a five-point Likert scale was primarily used. This scale ranged from very positive (5 points) to very negative (1 point) to measure the level of positivity. The cut-off value for attitude dimension was set at: 10–20 for negative attitude, 21–30 for neutral attitude, and 31–50 for positive attitude. Similarly, the cut-off value for practice dimension was set at: 15–30 for inactive practice, 31–45 for moderate practice, and 46–75 for proactive practice.

All the patients included in this study were diagnosed with psoriasis according to the diagnostic criteria for psoriasis. In cases where a patient has a lengthy disease duration, his or her historical medical history would be reviewed during the diagnostic process, and the diagnosis of psoriasis was established by taking their prior diagnoses, medical history, and an assessment of their skin lesions into account. In situations where there is no prior diagnosis or treatment history, and the skin lesions appear atypical, a biopsy would be performed for a definitive diagnosis. In cases where a patient had new skin lesions and exhibited typical locations and manifestations, a diagnosis of psoriasis could be made directly, while if the skin lesions appear atypical, a biopsy would be performed to confirm the diagnosis. The researchers recruited these confirmed patients through outpatient follow-up visits and patient WeChat groups. Participants who met the inclusion criteria were sent the questionnaire electronically. The online questionnaire based on the “*SoJump*”^1^ application of WeChat was used for the survey, and a QR code was generated to allow the data collection. Participants log in by scanning the QR code sent by WeChat, and then complete the questionnaire. To guarantee the quality and completeness of the questionnaire survey, each IP address could only submit the questionnaire once, and all questions in the questionnaire were mandatory. The completeness, internal coherence, and reasonableness of all questionnaires were checked by the investigators.

### Statistical analysis

Stata 17.0 (Stata Corporation, College Station, TX, USA) software was used for the statistical analysis. Descriptive analysis of the general characteristics of the participants and the KAP scores was performed as follow: continuous variables in normal distribution (KAP scores) were described by mean ± standard deviation (SD). Continuous variables that followed a normal distribution were compared using Student’s *t*-test or ANOVA, while those that did not were compared using the Mann–Whitney U test. Categorical variables including the demographic characteristics and answers to different question were described by *n* (%). Pearson’s correlation analysis was used to evaluate the relationship between the K, A, and P dimensions, with *r* < 0.4 is weak, *r* ≥ 0.4 < 0.6 is moderate, and ≥0.6 is strong. Structural equation modeling (SEM) was used to investigate path relationships between KAP and basic information. Two-sided *p* < 0.05 was considered statistically significant.

### Results

The questionnaire was distributed among 526 psoriasis patients, including 257 males (48.86%) psoriasis patients, with a 100% return rate. Their mean KAP scores were 8.09 ± 3.60 (possible range: 0–12), 31.94 ± 4.61 (possible range: 10–50), and 51.92 ± 8.83 (possible range: 15–75), respectively. Age was significantly linked to attitude (*p* < 0.001) and practice scores (*p* = 0.036), with females scoring higher. Education also associated with attitude scores (*p* < 0.001), with higher education levels correlating to higher scores. Employment showed significant associations with knowledge (*p* = 0.025) and practice scores (*p* = 0.912), with unemployed individuals scoring the highest in knowledge. Household income had a significant correlation with attitude scores (*p* = 0.009), where higher income levels were associated with higher scores. Marital status was significantly related to knowledge (*p* < 0.001) and practice (*p* = 0.923) scores, with divorced/widowed respondents having the highest knowledge scores. Parenthood also played a role, as individuals with children had higher knowledge (*p* < 0.001) and practice (*p* = 0.268) scores. Smoking showed significant association with attitude (*p* = 0.253) and practice (*p* = 0.002) scores, with never smokers having higher mean scores. Medical insurance type influenced with knowledge (*p* < 0.001) and practice (*p* = 0.529) scores, with individuals having commercial medical insurance scoring the lowest in knowledge. Psoriasis duration was significantly associated with knowledge and attitude scores (both *p* < 0.001), with individuals experiencing longer psoriasis duration scoring higher. Family history of psoriasis had a significant impact, with those having a family history psoriasis scoring higher in knowledge (*p* < 0.001) and attitude (*p* = 0.035). Additionally, taking medication for psoriasis was associated with higher knowledge (*p* < 0.001) and practice (*p* = 0.006) scores (Table 1).

**TABLE 1:** Demographic information.

Variables	*N* (%)	Knowledge score		Attitude score		Practice score	
		Mean ± SD	*p*-Value	Mean ± SD	*p*-Value	Mean ± SD	*p*-Value
**Total**	526 (100)	8.09 ± 3.60		31.94 ± 4.61		51.92 ± 8.83	
**Gender**			0.371		<0.001		0.036
Male	257 (48.86)	7.95 ± 3.73		31.23 ± 4.74		51.09 ± 8.40	
Female	269 (51.14)	8.23 ± 3.47		32.61 ± 4.39		52.71 ± 9.17	
**Age**	34.18 ± 10.48						
**Education**			0.778		<0.001		0.142
Middle school and below	84 (15.97)	8.14 ± 3.01		29.95 ± 4.86		53.54 ± 7.83	
High school	103 (19.58)	7.79 ± 3.83		32.42 ± 4.23		50.52 ± 8.99	
Bachelor’s degree	280 (53.23)	8.21 ± 3.68		32.36 ± 4.42		51.99 ± 8.81	
Master’s degree and above	59 (11.22)	7.98 ± 3.60		31.92 ± 5.12		51.69 ± 9.73	
**Ethnicity**			0.313		0.313		0.424
Han	483 (91.83)	8.14 ± 3.57		32.00 ± 4.56		52.01 ± 8.75	
Ethnic minority	43 (8.17)	7.56 ± 3.96		31.26 ± 5.21		50.88 ± 9.72	
**Employment**			0.025		0.082		0.912
Employed	314 (59.70)	7.94 ± 3.73		32.16 ± 4.73		51.87 ± 9.10	
Unemployed	44 (8.37)	9.20 ± 3.11		32.05 ± 3.85		50.89 ± 8.31	
Retired	17 (3.23)	8.76 ± 2.82		28.65 ± 4.82		51.76 ± 7.77	
Individuals	38 (7.22)	9.34 ± 2.93		32.13 ± 4.13		51.42 ± 6.05	
Housewife	29 (5.51)	7.79 ± 3.08		31.69 ± 4.41		52.52 ± 7.14	
Other	84 (15.97)	7.45 ± 3.71		31.71 ± 4.65		52.69 ± 9.91	
**Household income**			0.704		0.009		0.299
<2,000	81 (15.40)	7.85 ± 3.49		30.57 ± 4.97		53.10 ± 8.91	
2,000–5,000	176 (33.46)	8.23 ± 3.63		31.96 ± 4.73		51.32 ± 9.01	
5,000–10,000	133 (25.29)	8.31 ± 3.33		32.48 ± 3.88		52.66 ± 7.47	
10,000–20,000	58 (11.03)	8.07 ± 3.91		31.26 ± 4.83		52.10 ± 8.91	
>20,000	78 (14.83)	7.77 ± 3.87		32.88 ± 4.66		50.63 ± 10.25	
**Marital status**			<0.001		0.083		0.923
Married	294 (55.89)	8.64 ± 3.37		32.19 ± 4.55		52.02 ± 8.51	
Unmarried	200 (38.02)	7.16 ± 3.83		31.41 ± 4.79		51.85 ± 9.34	
Divorced/widowed	32 (6.08)	8.88 ± 2.89		32.94 ± 3.68		51.41 ± 8.66	
**Parenthood**			<0.001		0.532		0.268
No	240 (45.63)	7.48 ± 3.86		31.80 ± 4.76		51.45 ± 9.52	
Yes	286 (54.37)	8.60 ± 3.29		32.05 ± 4.49		52.31 ± 8.20	
**Smoking**			0.511		0.253		0.002
Never	349 (66.35)	8.05 ± 3.61		32.09 ± 4.66		52.80 ± 9.01	
Often	71 (13.50)	7.79 ± 3.68		31.10 ± 4.36		51.46 ± 7.90	
Continue	106 (20.15)	8.41 ± 3.52		31.99 ± 4.59		49.31 ± 8.33	
**Drinking**			0.868		0.441		0.227
Never	314 (59.70)	8.14 ± 3.62		31.99 ± 4.67		52.26 ± 9.31	
Often	104 (19.77)	8.12 ± 3.31		31.46 ± 4.76		52.23 ± 7.45	
Continue	108 (20.53)	7.93 ± 3.82		32.25 ± 4.31		50.61 ± 8.55	
**Medical insurance**			<0.001		0.059		0.529
Social medical insurance	419 (79.66)	8.26 ± 3.46		31.97 ± 4.60		51.96 ± 8.75	
Commercial medical insurance	7 (1.33)	3.86 ± 3.58		27.86 ± 4.91		53.57 ± 8.68	
Both social and commercial medical insurance	65 (12.36)	8.23 ± 3.82		32.54 ± 4.65		52.48 ± 8.90	
No insurance	35 (6.65)	6.60 ± 4.08		31.23 ± 4.33		49.97 ± 9.75	
**Psoriasis duration**			<0.001		<0.001		0.531
≤1 year	244 (46.48)	6.16 ± 4.01		31.00 ± 4.98		51.71 ± 10.56	
1–3 years	54 (10.29)	8.26 ± 2.68		30.85 ± 4.92		53.19 ± 8.19	
≥3 years	227 (43.24)	10.12 ± 1.71		33.20 ± 3.77		51.82 ± 6.70	
**Underlying or chronic diseases (multiple choice)**							
Hypertension	66 (12.55)	–		–		–	
Hyperlipidemia	50 (9.51)	–		–		–	
Diabetes	24 (4.56)	–		–		–	
Tumor	6 (1.14)	–		–		–	
Digestive system disorders	37 (7.03)	–		–		–	
Atheromatous plaque	9 (1.71)	–		–		–	
Depression and other psychological disorders	51 (9.70)	–		–		–	
Other	225 (42.78)	–		–		–	
Unwilling to disclose	155 (29.47)	–		–		–	
**Family history of psoriasis**			<0.001		0.035		<0.001
No	312 (59.32)	8.14 ± 3.54		31.90 ± 4.43		52.24 ± 8.60	
Yes	144 (27.38)	9.30 ± 2.51		32.56 ± 4.74		52.99 ± 6.87	
Unwilling to disclose	70 (13.31)	5.39 ± 4.29		30.83 ± 4.98		48.26 ± 12.06	
**Medication**			<0.001		0.024		0.006
No	232 (44.11)	6.58 ± 4.23		31.43 ± 4.67		50.74 ± 10.60	
Yes	294 (55.89)	9.29 ± 2.42		32.34 ± 4.54		52.85 ± 7.01	

**FIGURE 1:**
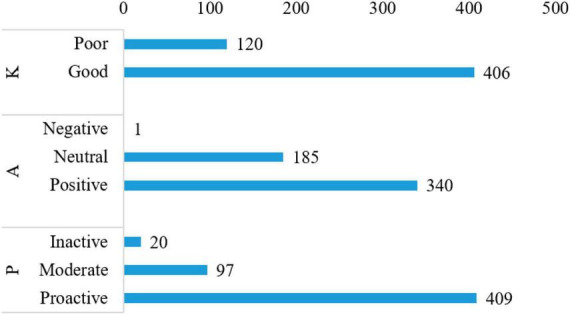
Knowledge, attitude, and practice (KAP) histogram. K (12 questions, 0–12 points with 0–6 as poor level of knowledge and 7–12 as good level of knowledge); A (10 questions with 10–20 as negative attitude, 21–30 as neutral attitude, and 31–50 as positive attitude); and P (15 questions with 15–30 as inactive practice, 31–45 as moderate practice, and 46–75 as proactive practice).

Knowledge, attitude, and practice histogram shows that 120 (22.8%) respondents had poor and 406 (77.2%) respondents had good knowledge of psoriasis. Regarding attitude toward psoriasis, only 1 (0.2%) respondent had a negative attitude, 185 (35.2%) had a neutral attitude, and 340 (64.6%) had a positive attitude. Among all respondents, 20 (3.8%) had had an inactive practice, 97 (18.4%) had a moderate practice, and 409 (77.6%) had a proactive practice (Figure 1). The results for the distribution of correct, incorrect, and unclear answers for questions K1–K12 showed that knowledge dimensions have higher percentages of correct answers, while attitude and practice dimensions have higher percentages of incorrect or unclear answers. K1 and K2 have high percentages of correct answers (78.14% and 80.99%, respectively), with a small percentage of incorrect answers (5.32% and 4.18%, respectively) and unclear answers (16.54% and 14.83%, respectively). K3 also showed a similar pattern with 74.71% correct answers, 5.32% incorrect answers, and 19.96% unclear answers. K4 stood out with a low percentage of correct answers (20.15%), a majority of incorrect answers (57.79%) and a smaller percentage of unclear answers (22.05%). K5, K9, and K10 showed relatively high percentages of correct answers (76.24%, 78.9%, and 79.66%, respectively) with similar percentages of incorrect and unclear answers. K6, K7, and K12 showed lower percentages of correct answers, ranging from 21.86% to 31.37%, and higher percentages of incorrect and unclear answers. K8 and K11 showed higher percentages of correct answers compared to K6, K7, and K12, with percentages ranging from 65.02% to 78.52% (Table 2).

**TABLE 2:** Distribution of question answered for knowledge dimension, *n* (%).

Items	Correct	Incorrect	Unclear
K1. Psoriasis is a chronic scaly skin disease, also known as “lepra alphos.”	411 (78.14)	28 (5.32)	87 (16.54)
K2. The appearance of red spots on the skin covered with silvery-white scales should be alerted to psoriasis.	426 (80.99)	22 (4.18)	78 (14.83)
K3. Psoriasis is caused by environmental influences on the basis of genetic factors.	393 (74.71)	28 (5.32)	105 (19.96)
K4. Psoriasis is not associated with the immune system.	106 (20.15)	304 (57.79)	116 (22.05)
K5. Psoriasis may be triggered by viral infections, stress, obesity, smoking, skin injuries, and some medications.	401 (76.24)	30 (5.7)	95 (18.06)
K6. Psoriasis is not easy to reoccur after being cured. (Psoriasis has the possibility of recurrence.)	165 (31.37)	264 (50.19)	97 (18.44)
K7. Psoriasis does not cause complications.	115 (21.86)	268 (50.95)	143 (27.19)
K8. Proper sunlight exposure can improve psoriasis.	342 (65.02)	62 (11.79)	122 (23.19)
K9. To avoid scratching and causing infection, the desquamation of scales in psoriasis should not be forced off by hand.	415 (78.9)	33 (6.27)	78 (14.83)
K10. Climate has a great impact on psoriasis, and cold and dry weather will aggravate the disease.	419 (79.66)	22 (4.18)	85 (16.16)
K11. Emotional fluctuations, sleep quality will aggravate psoriasis.	413 (78.52)	17 (3.23)	96 (18.25)
K12. Infection with COVID-19 does not have a negative impact on psoriasis.	126 (23.95)	199 (37.83)	201 (38.21)

The results on the distribution of responses for questions A1–A12 showed that the majority of patients have a positive or very positive attitude toward psoriasis, with 53.42% of patients answering in the very positive category and 16.73% answering in the positive category for question A1. However, there is also a significant number of patients with negative or very negative attitudes, with 40.87% of patients answering in the negative category for question A4 and 4.75% answering in the very negative category for question A5. For other questions, there is a mix of responses, with some patients indicating a very positive attitude and others indicating a negative attitude. While the majority of patients have a positive attitude toward psoriasis, there is also a significant proportion who hold negative attitudes (Table 3).

**TABLE 3:** Distribution of question answered for attitude dimension, *n* (%).

Items	Very positive	Positive	Neutral	Negative	Very negative
A1. Psoriasis makes me feel very unconfident and afraid of people laughing or commenting on my skin.	281 (53.42)	114 (21.67)	84 (15.97)	17 (3.23)	30 (5.7)
A2. I think psoriasis is difficult to prevent and therefore don’t care much about it.	88 (16.73)	71 (13.5)	142 (27)	148 (28.14)	77 (14.64)
A3. Although I think that psoriasis is difficult to prevent, the triggers for disease recurrence and aggravation should be avoided.	250 (47.53)	146 (27.76)	90 (17.11)	16 (3.04)	24 (4.56)
A4. I think psoriasis is a common skin disease that does not cause any complications.	67 (12.74)	42 (7.98)	118 (22.43)	215 (40.87)	84 (15.97)
A5. I think patients with psoriasis need psychological care from their families and society.	272 (51.71)	134 (25.48)	81 (15.4)	14 (2.66)	25 (4.75)
A6. I think psoriasis is easy to treat and not easy to reoccur.	54 (10.27)	35 (6.65)	109 (20.72)	202 (38.4)	126 (23.95)
A7. I think that after being cured of psoriasis, it is not necessary to have regular check-ups.	65 (12.36)	43 (8.17)	86 (16.35)	233 (44.3)	99 (18.82)
A8. I think daily care for psoriasis is too troublesome and makes me feel anxious.	176 (33.46)	160 (30.42)	117 (22.24)	46 (8.75)	27 (5.13)
A9. I think that it can be inherited to my children if I marry a patient who carries the disease gene.	126 (23.95)	167 (31.75)	162 (30.8)	43 (8.17)	28 (5.32)
A10. I think the current financial cost of psoriasis treatment is unaffordable.	143 (27.19)	138 (26.24)	169 (32.13)	45 (8.56)	31 (5.89)

The results for the distribution of responses for the practice dimension of the questions P1–P15 showed that the highest percentage of respondents answered “very positive” for P6 (53.61%), P5 (46.01%), and P8 (44.68%), whereas the lowest percentage of respondents chose “very positive” for P13 (21.86%), P14 (24.33%), and P15 (23.57%). The highest percentage of respondents chose “very negative” for P12 (13.12%), P13 (16.73%), and P15 (14.45%), whereas the lowest percentage of respondents answered “very negative” for P10 (4.75%), P11 (6.27%), and P12 (5.13%). There was a notable occurrence of high percentages of “Neutral” responses such as for P2 (24.33%), P3 (28.52%), P4 (25.86%), P5 (17.49%), P7 (26.24%), P12 (24.9%), P13 (21.29%), P14 (32.32%), and P15 (27.19%), and “Negative” responses such as for P12 (19.01%), P13 (23.19%), P14 (11.98%), and P15 (15.59%) (Table 4).

**TABLE 4:** Distribution of question answered for practice dimension, *n* (%).

Items	Very positive	Positive	Neutral	Negative	Very negative
P1. Psoriasis makes me often avoid social occasions and public places in the summer.	225 (42.78)	121 (23)	91 (17.3)	40 (7.6)	49 (9.32)
P2. I would avoid irritating foods such as coffee, strong tea, and spices.	169 (32.13)	117 (22.24)	128 (24.33)	46 (8.75)	66 (12.55)
P3. I would be suffering from mental stress, which causes the disease to reoccur.	146 (27.76)	121 (23)	150 (28.52)	56 (10.65)	53 (10.08)
P4. I would improve my sleep and keep my mood.	182 (34.6)	145 (27.57)	136 (25.86)	31 (5.89)	32 (6.08)
P5. I would stop smoking and drinking.	242 (46.01)	83 (15.78)	92 (17.49)	56 (10.65)	53 (10.08)
P6. I would keep my skin clean.	282 (53.61)	146 (27.76)	57 (10.84)	14 (2.66)	27 (5.13)
P7. I can do proper sports to improve my physical fitness and strengthen my immune systems.	182 (34.6)	127 (24.14)	138 (26.24)	45 (8.56)	34 (6.46)
P8. I would change bed sheet in time and keep the bed clean and free of crumbs.	235 (44.68)	170 (32.32)	77 (14.64)	18 (3.42)	26 (4.94)
P9. I would wear loose, soft clothing to minimize the irritation to my skin.	221 (42.02)	165 (31.37)	85 (16.16)	27 (5.13)	28 (5.32)
P10. I would cut my nails short to avoid scratching the skin lesions site.	235 (44.68)	160 (30.42)	83 (15.78)	23 (4.37)	25 (4.75)
P11. I would follow the doctor’s advice to take active treatment and make regular follow-up visits.	217 (41.25)	157 (29.85)	85 (16.16)	34 (6.46)	33 (6.27)
P12. My disease has not recurred after sun exposure and hot springs bath.	139 (26.43)	87 (16.54)	131 (24.9)	100 (19.01)	69 (13.12)
P13. My disease has not recurred after topical or oral Chinese herbal medicine.	115 (21.86)	89 (16.92)	112 (21.29)	122 (23.19)	88 (16.73)
P14. I would replicate treatments that worked for other patients but it failed to work for me.	128 (24.33)	112 (21.29)	170 (32.32)	63 (11.98)	53 (10.08)
P15. I would miss the classes/attendance due to my treatment.	124 (23.57)	101 (19.2)	143 (27.19)	82 (15.59)	76 (14.45)

**FIGURE 2:**
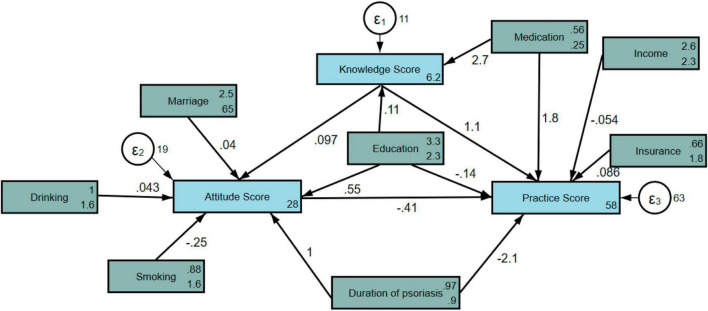
Illustration of structural equation model (SEM) analysis.

Pearson’s correlation analysis showed a significant weak positive correlation between knowledge and attitude (*r* = 0.186, *p* < 0.001). A significant moderate positive correlation between knowledge and practice (*r* = 0.313, *p* < 0.001). The attitude and practice showed a significantly weak negative relationship (*r* = −0.181, *p* < 0.001) (Table 5). Moreover, SEM showed that medication (β = 2.74, 95% CI: 2.17, 3.32, *p* < 0.001) has significantly positive effect on knowledge. Education (β = 0.56, 95% CI: 0.31, 0.81, *p* < 0.001) and duration of psoriasis (β = 1.01, 95% CI: 0.54, 1.49, *p* < 0.001) have significantly positive effect on attitude. Knowledge (β = 1.03, 95% CI: 0.80, 1.26, *p* < 0.001) and medication (β = 4.59, 95% CI: 2.78, 6.40, *p* < 0.001) has significantly positive effect on practice, while attitude (β = −0.41, 95% CI: −0.57, −0.26, *p* < 0.001) and duration of psoriasis (β = −2.53, 95% CI: −3.49, −1.57, *p* < 0.001) exhibit significantly negative effect on practice (Table 6 and Figure 2).

**TABLE 5:** Results of Pearson’s correlation analysis.

	Knowledge	Attitude	Practice
Knowledge	1		
Attitude	0.186 (*p* < 0.001)	1	
Practice	0.313 (*p* < 0.001)	−0.181 (*p* < 0.001)	1

**TABLE 6:** Total effect of structural equation model (SEM) analysis.

	β (95% CI)	*p*-Value
**K**		
Education	0.11 (−0.08, 0.30)	0.247
Medication	2.74 (2.17, 3.32)	<0.001
**A**		
K	0.10 (−0.03, 0.22)	0.120
Marital status	0.04 (−0.01, 0.09)	0.099
Education	0.56 (0.31, 0.81)	<0.001
Psoriasis duration	1.01 (0.54, 1.49)	<0.001
Smoking	−0.25 (−0.60, 0.09)	0.148
Drinking	0.04 (−0.29, 0.38)	0.804
Medication	0.27 (−0.07, 0.61)	0.125
**P**		
K	1.03 (0.80, 1.26)	<0.001
A	−0.41 (−0.57, −0.26)	<0.001
Marital status	−0.02 (−0.04, 0.004)	0.115
Education	−0.26 (−0.78, 0.27)	0.337
Psoriasis duration	−2.53 (−3.49, −1.57)	<0.001
Smoking	0.10 (−0.04, 0.25)	0.163
Drinking	−0.02 (−0.16, 0.12)	0.804
Medical insurance	0.09 (−0.43, 0.60)	0.744
Income	−0.05 (−0.54, 0.43)	0.826
Medication	4.59 (2.78, 6.40)	<0.001

## Discussion

This study found that psoriasis patients have good knowledge, positive attitude, and proactive practice toward the disease. Education, medication, duration of psoriasis might have effect on their KAP. The findings of this study suggest that interventions to improve education and access to medication could lead to better disease management and outcomes.

This study found positive correlations between knowledge and attitude, and knowledge and practice, and a negative correlation between attitude and practice in psoriasis patients. The analysis of demographic characteristics revealed that several factors were significantly associated with KAP scores. Education was associated with attitude scores, with higher education levels associated with higher mean scores. This finding aligns with a previous study that has shown a positive correlation between education level and health-related knowledge and attitudes (15). The KAP histogram also showed that education and medication were significantly associated with knowledge scores about psoriasis, with higher education levels leading to greater knowledge. This finding is consistent with previous research showing the role of education in increasing knowledge about medical conditions (16). Medication was also significantly associated with practice scores, with individuals on medication having higher mean scores. This finding is expected, as individuals who are actively using medication for their condition are likely to have a better understanding of the disease and be more compliant with treatment recommendations (17). SEM analysis also found that education and medication had a positive relationship with knowledge about psoriasis, with those having higher levels of education and were on medication exhibited greater knowledge. Household income was significantly associated with attitude scores, with higher income levels associated with higher mean scores. Regarding the practice of psoriasis management, household income showed a negative relationship. Higher household incomes were associated with greater access to resources and healthcare services for managing psoriasis effectively, while those without medical insurance barriers in accessing necessary treatments. These findings are consistent with previous research highlighting the impact of socioeconomic factors on psoriasis management and treatment outcomes (18, 19). Household income and medical insurance were identified as predictors of practice, with both being positively associated with psoriasis practice. Marital status was significantly associated with knowledge and practice scores, with divorced/widowed respondents having the highest mean scores in both categories. Marital status showed a borderline significant association with attitude scores, suggesting it may have some influence on attitudes toward psoriasis. Married individuals and those who reported drinking and smoking, had more positive attitudes toward psoriasis. Smoking was significantly associated with attitude and practice scores, with never smokers having higher mean scores. This finding is consistent with previous research that has shown that smoking is associated with poor health outcomes and lower adherence to treatment regimens (20). This may be due to the fact that individuals who are no longer in a committed relationship may have more time and autonomy to focus on managing their condition (21). This could be further attributed to the social support and understanding within marital relationships (21), and alcohol and tobacco use possibly serving as coping mechanisms or sources of stress relief, influencing attitudes toward psoriasis (20). Medical insurance type was significantly associated with knowledge and practice scores, with individuals with commercial medical insurance having the lowest mean scores. This may be due to

differences in access to healthcare services and resources between different types of insurance. However, there was no significant association between medical insurance type and practice scores, suggesting that insurance type may not have a significant impact on actual behaviors. These results are consistent with a previous study (22). The psoriasis duration (i.e., duration of the disease) was significantly associated with knowledge and attitude scores, with individuals with a longer psoriasis duration having higher mean scores. Psoriasis duration was significantly associated with attitude scores, with longer duration possibly leading to potentially less positive attitude due to increased challenges and negative impacts. This may be due to the fact that individuals with a longer disease duration have had more time to acquire knowledge about their condition and develop a positive attitude toward managing it. Overall, these findings emphasize the importance of considering individual characteristics and circumstances in managing and educating psoriasis patients. Understanding these associations can help healthcare providers tailor interventions and support to meet the specific needs of different patient groups effectively. The study found positive correlations between knowledge and attitude, as well as between knowledge and practice, indicating that individuals with more knowledge about psoriasis are more likely to have positive attitudes and engage in related practices. However, there was a negative correlation between attitude and practice, suggesting that individuals with positive attitudes may not always translate those attitudes into action. These findings are consistent with previous research on KAP scores in other dermatological diseases, such as acne vulgaris (23) and atopic dermatitis (24), which also showed positive correlation between knowledge and attitudes or behavior change. The positive correlation between knowledge and attitude may be attributed to the information-processing theory, suggesting that greater knowledge leads to more positive attitudes (25). Additionally, individuals with positive attitudes might actively seek out more information, contributing to their greater knowledge. Conversely, the negative correlation between attitude and practice may be influenced by various factors like social norms, peer influence, or lack of self-efficacy. Both knowledge and attitude were significantly associated with practice scores, indicating that individuals with higher knowledge and more positive attitudes had more practice approaches toward managing psoriasis.

All respondents possessed a relatively good level of knowledge about psoriasis, but their attitude and practice scores were low. This aligns with previous research, which also found that patients had adequate knowledge of psoriasis basics but lacked understanding of self-management and psychosocial aspects (26). These results underscore the need for comprehensive education and support for psoriasis patients, particularly in areas where knowledge is lacking. Targeted interventions should focus on improving attitudes and promoting effective self-care practices to enhance overall management and wellbeing of patients. The respondents exhibited a range of attitudes toward psoriasis, most of them having positive or very positive views. However, a notable proportion also had negative attitudes. This supports previous research showing that patients with psoriasis often experience negative emotions, social exclusion, and stigmatization (27–29). However, other studies have reported positive attitudes among patients who actively seek treatment and engage in self-care practices, leading to better quality of life (30, 31). The presence of both positive and negative attitudes highlights the complexity of the patient experience, emphasizing the importance of individualized care and support to address negative attitudes or emotions. Patient-centered care is crucial in addressing the psychosocial aspects of psoriasis and promoting positive attitudes. The respondents showed varying responses, with some practices receiving strong positive attitudes while others elicited weaker positive or even negative attitudes. These results emphasize the need to consider the diverse attitudes toward different practices when designing interventions or strategies for managing psoriasis. Identifying areas that require more attention and support can lead to more effective and tailored approaches in psoriasis management.

This study has a few limitations. First, this study was conducted in Xi’an, Shaanxi, China, which may limit the generalizability of the findings to other populations or countries. Additionally, in this study, since patients in this study were recruited in the dermatology outpatient department of Shaanxi Provincial People’s Hospital, who were more likely to be urban residents, so we did not investigate regional information, and could not compare the KAP scores differences among different regions. We plan to conduct similar surveys in more hospitals, including primary hospitals, to address this limitation. Secondly, due to the cross-sectional nature of the study, the data was collected at a single time point, thus limiting the ability to establish causality or examine changes over time. Third, the study design does not provide psychometric properties or formal validation of the questionnaire. Fourth, the study lacks a comparison group of individuals without psoriasis, thus lacking a comparison of the KAP scores of psoriasis patients with a control group to identify unique factors specific to psoriasis patients.

## Conclusion

In conclusion, psoriasis patients have good knowledge, positive attitude, and proactive practice toward the disease. Education, medication, duration of psoriasis might have effect on their KAP. The findings of this study underscore the importance of education, medication, psoriasis duration, and KAP scores in understanding and management of psoriasis among individuals, suggesting that there is a need for further interventions to improve education and access to medication for better management of the disease.

## Data availability statement

The original contributions presented in this study are included in this article/supplementary material, further inquiries can be directed to the corresponding author.

## Ethics statement

The studies involving humans were approved by the Ethics Committee of Shaanxi Provincial People’s Hospital (2023006). The studies were conducted in accordance with the local legislation and institutional requirements. The participants provided their written informed consent to participate in this study.

## Author contributions

JT: Formal analysis, Writing – original draft. LZ: Methodology, Writing – original draft. XZ: Formal analysis, Writing – original draft. LY: Conceptualization, Formal analysis, Writing – review & editing.

## Footnotes

1
https://www.wjx.cn/
